# Dispersionless Manipulation of Reflected Acoustic Wavefront by Subwavelength Corrugated Surface

**DOI:** 10.1038/srep10966

**Published:** 2015-06-16

**Authors:** Yi-Fan Zhu, Xin-Ye Zou, Rui-Qi Li, Xue Jiang, Juan Tu, Bin Liang, Jian-Chun Cheng

**Affiliations:** 1 Key Laboratory of Modern Acoustics, MOE, Institute of Acoustics, Department of Physics, Nanjing University, Nanjing 210093, P. R. China; 2 Collaborative Innovation Center of Advanced Microstructures, Nanjing University, 210093, P. R. China; 3 State Key Laboratory of Acoustics, Chinese Academy of Sciences, Beijing 100190, P. R. China

## Abstract

Free controls of optic/acoustic waves for bending, focusing or steering the energy of wavefronts are highly desirable in many practical scenarios. However, the dispersive nature of the existing metamaterials/metasurfaces for wavefront manipulation necessarily results in limited bandwidth. Here, we propose the concept of dispersionless wavefront manipulation and report a theoretical, numerical and experimental work on the design of a reflective surface capable of controlling the acoustic wavefront arbitrarily without bandwidth limitation. Analytical analysis predicts the possibility to completely eliminate the frequency dependence with a specific gradient surface which can be implemented by designing a subwavelength corrugated surface. Experimental and numerical results, well consistent with the theoretical predictions, have validated the proposed scheme by demonstrating a distinct phenomenon of extraordinary acoustic reflection within an ultra-broad band. For acquiring a deeper insight into the underlying physics, a simple physical model is developed which helps to interpret this extraordinary phenomenon and predict the upper cutoff frequency precisely. Generations of planar focusing and non-diffractive beam have also been exemplified. With the dispersionless wave-steering capability and deep discrete resolution, our designed structure may open new avenue to fully steer classical waves and offer design possibilities for broadband optical/acoustical devices.

The recent decade has witnessed a rapid development in the field of wavefront manipulation research^1^,^2^,^3^,^4^,^5^,^6^,^7^,^8^,^9^,^10^,^11^,^12^,^13^,^14^,^15^,^16^,^17^,^18^,^19^. In conventional optical and acoustic components, the wavefront shaping is achieved on the basis of reflection, refraction or diffraction of waves. The emergence of metasurfaces provides a new way to manipulate wavefront freely by introducing abrupt changes of optical/acoustical phases, which breaks the dependence on the propagation effect, and enables molding wavefront into arbitrary shapes with subwavelength resolution^2^. Optical metasurfaces have exhibited great potentials to achieve novel phenomena such as extraordinary refraction/reflection^3^,^4^,^5^,^6^, coupling of propagating wave into evanescent waves^7^, planar focusing^8^,^9^,^10^, non-diffracting beams^11^, and vortex^12^. Acoustic counterparts have also been realized with similar capability of acoustic wavefront manipulation^13^,^14^,^15^,^16^,^17^,^18^,^19^. Despite the tremendous prospect of these devices with extraordinary wave manipulation flexibilities, they are inevitably frequency dependent and have limited bandwidth, in which the desired phase response of a subunit is designed only for a specific frequency and, moreover, the transverse size of a supercell needs to be related to wavelength^1^,^2^,^3^,^4^,^5^,^6^,^7^,^8^,^9^,^10^,^11^,^12^,^13^,^14^,^15^,^16^,^17^,^18^,^19^. As a result, the change of frequency will unavoidably lead to different phase profiles. So far, the manipulation of optic/acoustic waves with unlimited bandwidth is still challenging and remains an open question.

Here, we propose the concept of dispersionless wavefront manipulation and develop a general scheme to design reflective surface with a hitherto inaccessible functionality of going beyond the restriction of bandwidth. A specific implementation by subwavelength corrugated surface (SCS) has been demonstrated theoretically and experimentally to mimic the desired gradient profile, both showing the distinct property of extraordinary reflection in an ultra-broad band. A simple model based on the phased array theory is presented to interpret this extraordinary phenomenon. By applying the same design method, more complicated phase profiles can be realized via the gradient surface with subwavelength resolution. We anticipate our designed structure with distinct mechanism will constitute a significant step towards practicable wavefront-controlling devices with broadband functionalities and have enlightening significance in the field of optical and acoustical wavefront engineering.

## Results

### Concept of dispersionless phase front

For the purpose of realizing dispersionless wavefront manipulation, we start from the fundamental difficulty of eliminating the frequency dependence in generalized law of reflection, which is deduced by Fermat’s principle and generally governs the behavior of the reflected wave at surface with a spatially-varying phase abrupt change introduced, as follows^2^
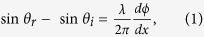
where *θ*_*r*_ and *θ*_*i*_ are the angles of reflection and incidence, respectively. *λ* is wavelength and *ϕ* *=* *ϕ(x)* is the phase response at the surface. The reflected angle *θ*_*r*_ can be calculated as
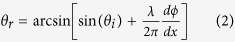


It is expected from Eq. (2) that various phase fronts can be yielded by designing the function of *dϕ/dx* appropriately. For instance, one can realize extraordinary reflection by choosing *dϕ/dx* as a constant everywhere on the interface, while planar focusing requires a specific *dϕ/dx* that varies with *x* to control the convergence of wave. Actually, these are the common ways the wavefront manipulations are achieved in previous designs. However, the optic/acoustic elements employed for realizing the desired phase gradient, are usually frequency selective, leading to the wavelength-dependence of the term *dϕ/dx*. Then the term (*λ/2π*)(*dϕ/dx)* seems to be necessarily a function of the incident wavelength, which means *θ*_*r*_ must differ at different frequencies^1^,^2^,^3^,^4^,^5^,^6^,^7^,^8^,^9^,^10^,^11^,^12^,^13^,^14^,^15^,^16^,^17^,^18^,^19^. As a result, the conventional wavefront-manipulation devices always have dispersion characteristics and limited bandwidth. Although some efforts have been dedicated to the design of broadband devices, their bandwidth is broadened by simply assuming an *dϕ/dx* invariant with respect to frequency, making it impossible to realize a constant reflected angle for real broadband signals^6^,^9^.

Observation of Eq. (2) suggests, however, that it is quite possible to exploit a frequency-dependent but appropriately-designed phase profile, i.e., *dϕ/dx*, to compensate the dispersion in the factor (*λ/2π*) perfectly, rather than leading to a highly dispersive wavefront manipulation as expected conventionally. For making the term (*λ/2π*)(*dϕ/dx)* independent of frequency, the phase gradient *dϕ*/*dx* should have the following form
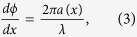
where *a*(*x*) is a function of *x* and independent of *λ*.

**Figure 1:**
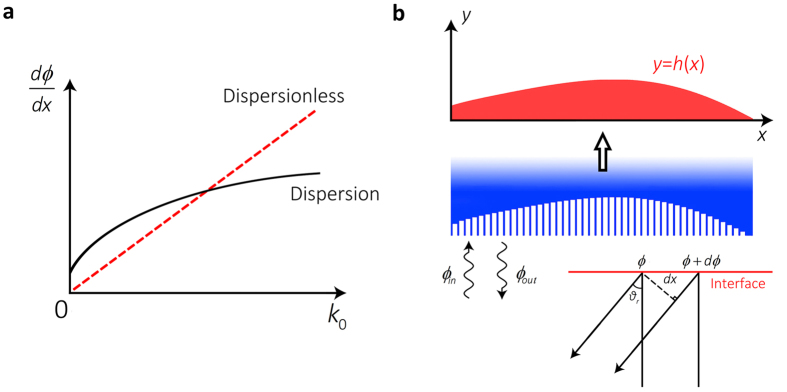
Schematic illustration of dispersionless SCS. (**a**) Schematic illustration of dispersionless phase front. A dispersionless phase front should have *dϕ*/*dx* proportional to *k*_0_. (**b**) Schematic illustration of SCS made of grooves with subwavelength width below the surface. Blue region is filled with acoustically rigid medium and the background medium is chosen as air. The depth of grooves is a function of *x*, i.e., *y* *=* *h*(*x*), which determines the spatially-varying phase shift between incidence and reflection.

The above analyses reveal the potential to realize dispersionless wavefront manipulation via design of a specific gradient surface. Figure 1(a) gives the schematic illustration of dispersionless phase front, which shows that the desired phase gradient *dϕ/dx* should be proportional to the wave number *k*_0_ *=* *2π/λ*, i.e., *ϕ*(*x*) *=* *k*_0_∫*a*(*x*)*dx*. Apparently, the presence of the factor of *k*_0_ is for vanishing the frequency dependence, while the remaining term (*a*(*x*)) that depends on *x* exclusively, can still be tailored freely to yield arbitrary wavefront, as will be discussed later.

In the following, we show how the SCS allows arbitrary control over the reflected phase front with the specific condition given by Eq. (3) being satisfied. Figure 1(b) shows the schematic of SCS. By decorating the surface with grooves that has subwavelength width, the phase of the wave is delayed when propagating along a round trip in each groove. Compared with a normal flat surface, the decorated one adds a local phase shift to the reflected wave. The relationship between input and output phases is simply determined by the wave path, as follows
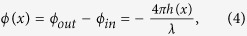
where *h*(*x*) is the depth of groove, and *ϕ*(*x*) represents the spatial phase response at the surface. In the proposed SCS structure, the dimension of the grooves along *x*-direction is subwavelength, which guarantees a subwavelength resolution of phase profile along the surface. On the other hand, the depth of the grooves along *y*-direction is comparable to the working wavelengths in order to achieve enough phase retardation. Thus, by choosing the depths of the grooves at each spatial position differently, spatially-varying phase shift can be obtained for the reflected wave. It is noteworthy that the phase response given by Eq. (4) agrees with Eq. (3) in form, indicating that the requirement for eliminating the frequency-related term is met excellently. The reflected wave direction can be deduced by Eq. (2)
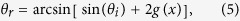
where *g*(*x*) *=* *dh*(*x*)/*dx* is the spatial gradient of the groove array. In such cases, the reflected direction is independence of wavelength and solely dominated by the choice of *g*(*x*). Thus, by designing *g*(*x*) appropriately, arbitrary phase front can be achieved in broad band, as will be demonstrated later.

### Ultra-broadband extraordinary reflection by SCS

**Figure 2:**
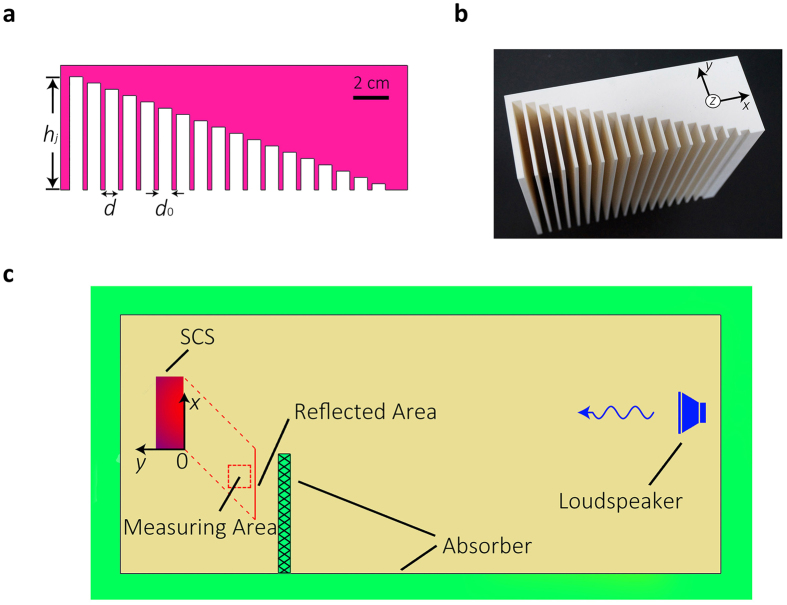
Schematic illustration of ERSCS and experimental setup. (**a**) Schematic cross-section of ERSCS made of 18 grooves, with period *d* *=* 1cm and width *d*_0_ *=* 0.75cm. The depth *h*_*j*_ of each groove increases from 3.535 mm to 63.63 mm with step of 3.535 mm. The size in *x*-*y* plane is 19.5 cm × 7 cm. (**b**) A photograph of the ERSCS sample made of acrylonitrile butadiene styrene (ABS) plastic manufactured by 3D printer. The size is 19.5 cm × 7 cm × 18 cm. (**c**) Schematic illustration of 2-D experimental system. The measuring area is a 6 cm × 6 cm square region centered at (-8 cm, -16 cm). The ‘Reflected Area’ is defined as a 19.5 cm wide cross profile with its center coordinate (−9.75 cm, −19.5 cm). An absorptive plate was placed between source and measuring region to separate the incident and reflected acoustic fields.

The following analyses take a particular extraordinary reflection SCS (ERSCS) with constant gradient of groove arrays (viz., *g*(*x*)) as example. Figure 2(a) shows the structural parameters of the ERSCS with *g* *=* 0.3535. For an incident wave with frequency of *f*_0_ *=* *c*_*0*_/2*h*_18_ *=* 2695 Hz (*c*_*0*_ *=* 343 *m/s* is sound speed in air), the range of the phase shift provided by the grooves is 0–2*π* with step of *π*/9. Therefore, for an incident wave with arbitrary frequency of *βf*_0_.(*β* *>* 0), the reflected phase can be also modulated linearly between 0–2*βπ* with step of *βπ/*9, which means the requirement for yielding the extraordinary reflection can always be fulfilled at different frequencies.

Figures 2(b,c) show the photograph of the sample and the 2-D schematic of the experimental system, respectively. The measurements were performed in the anechoic chamber. To obtain a plane wave, a loudspeaker was located 3 m away from the sample. The measuring area and the center of loudspeaker are located in the same *x-y* plane. The reflected angle deduced from Eq. (5) for normally-incident wave is 45°. An absorptive plate was placed in between to separate the incident and reflected acoustic fields. The error caused by diffraction effect near the edge of the plate should be negligible since both the sizes of ERSCS and the measuring area are much larger than the incident wavelength.

**Figure 3:**
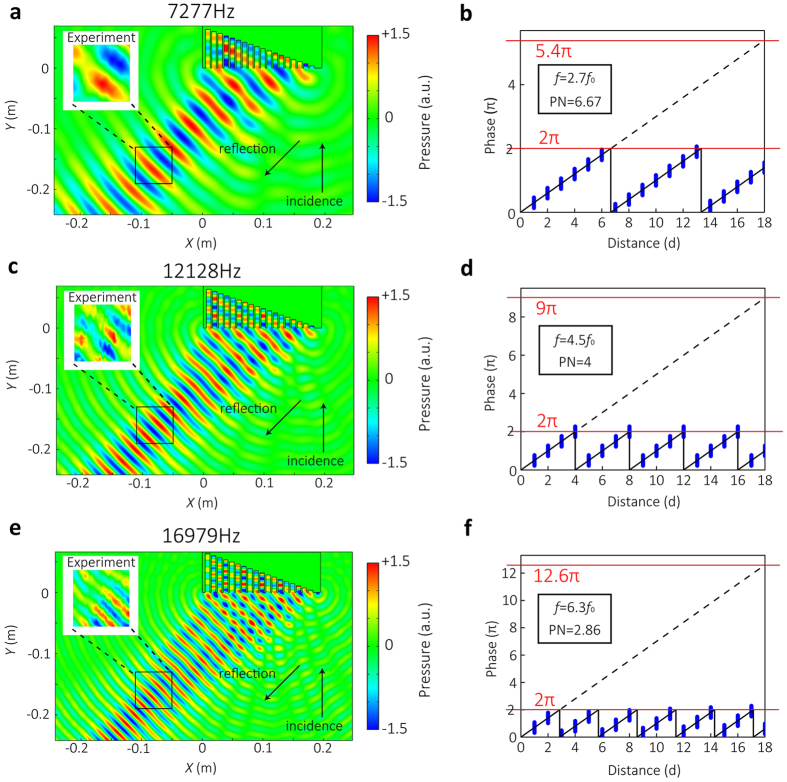
Numerical and experimental demonstrations of ultra-broadband extraordinary reflection by SCS. (**a**,**c**,**e**) Scattered acoustic pressure fields of ERSCS in simulation at 7.277 kHz, 12.128 kHz, and 16.979 kHz, respectively. The insets show the sound fields measured in the in the measuring area of Fig. 2(c). (b, d, f) The corresponding phase distributions along the interface at 7.277 kHz, 12.128 kHz, and 16.979 kHz, respectively. The phase keeps a linear dependence on the distance for different frequencies.

We have performed a series of measurements within a broadband, and the typical results will be presented as follows. Numerical simulations have also been carried out for comparison. The numerical and the experimental results are illustrated in Fig. 3 for three particular frequencies: 2.7*f*_0_, 4.5*f*_0_, and 6.3*f*_0_ (viz., 7.277 kHz, 12.128 kHz and 16.979 kHz). A parameter of phase number (PN), which represents the number of discrete phases within 2π range, is defined as PN *=* *n*/*β* to evaluate the resolution of the phase surface. For the three considered frequencies, the PN values are 6.67, 4, and 2.86, respectively. Good agreement is observed between the numerical and experimental results, in which the extraordinary reflection of 45°, exactly the same as the theoretical prediction, can be identified within an ultra-broadband frequency range. It is worth mentioning that reflected angle yielded by the proposed structure can be controlled freely within a remarkably wide range (approximately −80°–+80°). Note also that the effect of extraordinary reflection can be maintained in the cases of oblique incidence because the grooves with subwavelength width only permit zeroth-order mode to propagate, making the phase response independent of the incident direction. We have also performed a series of numerical simulations to estimate the effectiveness of the proposed structure for oblique incident waves and the numerical results have verified that this extraordinary phenomenon also occurs with high efficiency in oblique incidence cases. (See Supplementary Materials).

### Physical model based on phased array theory

**Figure 4:**
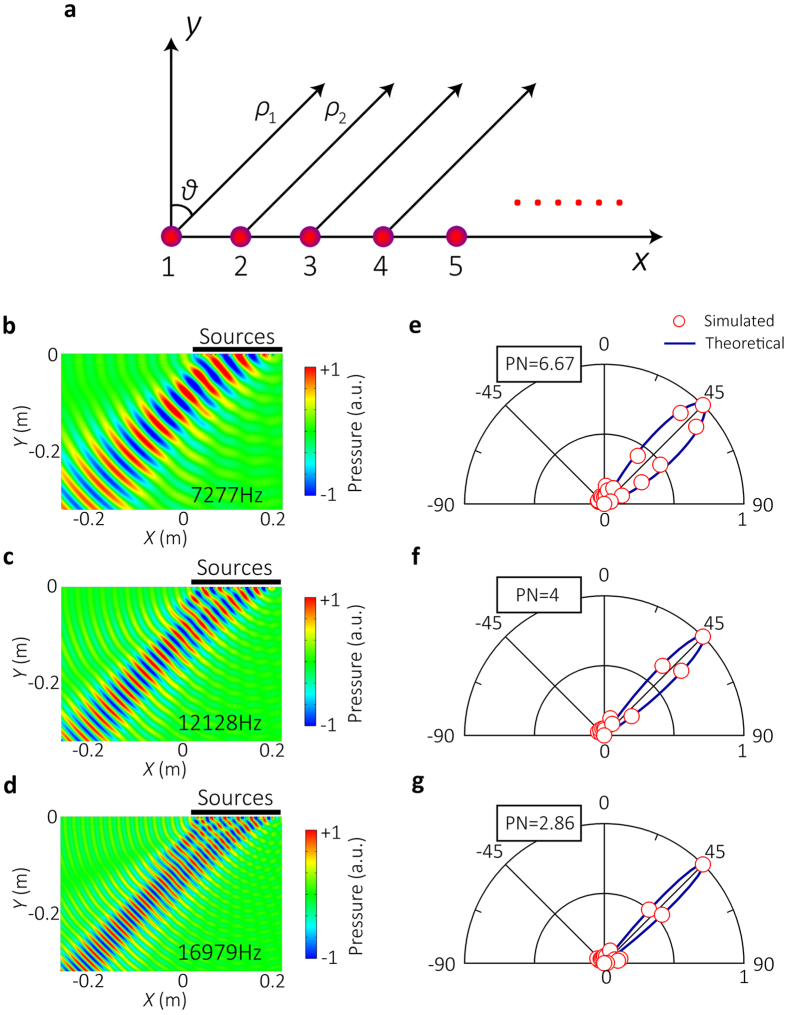
Physical model based on phased array theory. (**a**) Schematic illustration of the physical model based on phased array theory. The total acoustic pressure field is the sum of the secondary sources with different phase delays. (**b**, **c**, **d**) The acoustic pressure fields given by Eq. (6) at 7.277 kHz, 12.128 kHz, and 16.979 kHz, respectively. (e,f,g) The directional factors 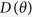 given by Eq. (9) (blue line) and the simulated ones (red circle) at 7.277 kHz, 12.128 kHz, and 16.979 kHz, respectively.

The simplicity in the structure of this particular ERSCS enables an analytical analysis for better understanding of its underlying mechanism, which can be accomplished by exploiting the phased array theory. At orifice of an individual groove, the incident wave will induce a vibration of air that behaves as a secondary radiation source for which the strength factor *A*_*j*_ is estimated approximately by *A*_*j*_ *=* *Sv*_*j*_, where *v*_*j*_ is normal velocity at the orifice and *S* is cross-section of each groove. Due to the subwavelength width of groove, we can simply consider the abnormal reflected wave as a new acoustic radiation by a line of secondary sources with phase delays modulated by the SCS with varying groove depths, as shown in Fig. 4(a). Assuming the distance between observation point and the *j*th source is *ρ*_*j*_, the total reflected pressure can be expressed as
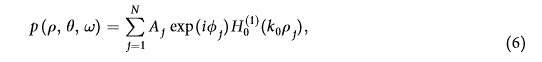
where 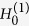 is zeroth order Hankel function of the first kind and *ϕ*_*j*_ is the phase delay of each secondary source. When the incident wave is a plane wave and width of each groove is same, all of the strength factors should be the same, i.e., *A*_*j*_ ≡ *A*. Since the phase delay by each groove is *ϕ*_*j*_ *=* 2*k*_0_*h*_*j*_ with *h*_*j*_ *=* (*N* − *j*)*dg*.*(N* *=* *n* + 1 *=* 19), at far-field Eq. (6) becomes
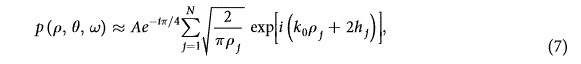
where *ρ*_*j*_ = *ρ*_1_ − ( *j* − 1)Δ*ρ* and 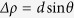,. The center of the source array is set as reference point and the distance is approximately *ρ* = *ρ*_1_ − *L*Δ*ρ*/2*d*, where *L* = (*N* − 1)*d* is the total length of line source array. Then one has
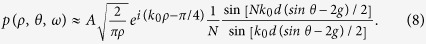


Equation (8) implies that the maximum of the radiation always appears at a particular angle, 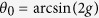, which is totally independent of *k*_0_. The field of the extraordinary reflected wave at 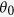 is 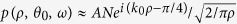. Then, Eq. (8) can be expressed as *p*(*ρ*, *θ*, *ω*) = *p*(*ρ*, *θ*_0_, *ω*)*D*(*θ*), where *D*(*θ*), is the directional factor, given as below
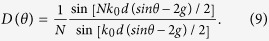


Figures 4(b–d) show the acoustic pressure fields given by Eq. (6) at different frequencies. Figures 4(e–g) show a comparison between the values of *D*(*θ*) given by Eq. (9) and by numerical simulation at corresponding frequencies. The theoretical results show that the extraordinary reflections appear at *θ* = 45° without sidelobes, which agree excellently with the numerical and experimental results.

### The bandwidth of ERSCS

**Figure 5:**
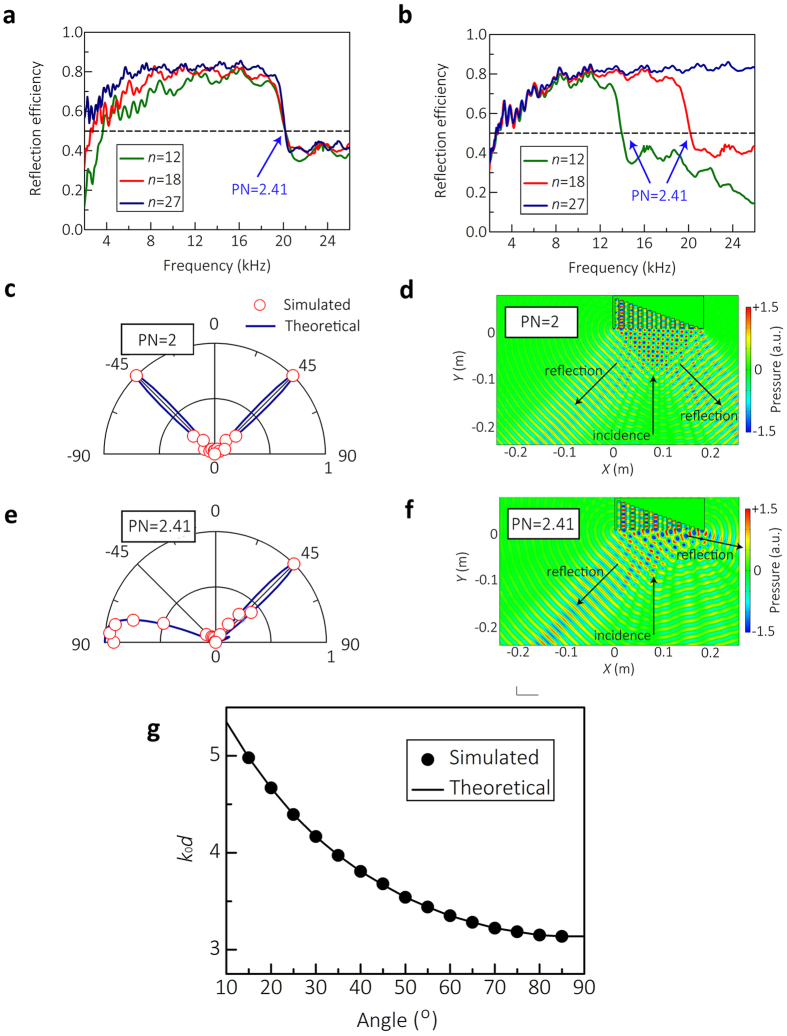
The bandwidth of ERSCS. The reflection efficiency (*R*) spectra with division numbers (*n*) of 12, 18, and 27 with the period *d* being held fixed in (a) and the total width of the ERSCS being constant in (b). (c,e) The direction factors 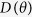 given by Eq. (9) (blue line) and the simulated ones (red circle) at PN = 2 and PN = 2.41 (corresponds to *K*_0_*d* = 3.68). (d,f) Numerical results of the scattered acoustic pressure field at PN = 2 and PN = 2.41. (g) The relationship between the values of 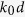 and the extraordinary reflection angles when the sidelobe appears at *θ*_0_ = −90°.

In principle, our scheme is capable of yielding a completely dispersionless wavefront manipulation. In practice, however, the desired gradient surface must be mimicked by a certain structure, which is necessarily frequency-dependent. Nevertheless, the operating bandwidth should be conveniently adjusted or extended according to the practical requirement. Therefore, it is important to investigate the influence of structural parameters on the efficiency and bandwidth of the ERSCS. We define a parameter of *R* *=* *W*/*W*_0_ with *W*_0_ and *W* being the sound power on the ERSCS and the ‘Reflect Area’ in Fig. 2(c) respectively, which can be used to quantitatively evaluate the performance of the ERSCS. Figures 5(a, b) show the reflection efficiency spectra simulated for three particular cases: *n* *=* 12, 18, and 27 respectively. For the ERSCS model with *n* *=* 18, the effective bandwidths, which is defined as *R* > 0.5, is from 2.5 to 20.1 kHz (about 3 octaves). With a constant period *d*, as shown in Fig. 5(a), the lower cutoff frequency (*f*_*cl*_) of the ERSCS decreases with increasing *n*, while the upper cutoff frequency (*f*_*ch*_) remains invariant. On the other hand, with a constant ERSCS width, *f*_*ch*_ shifts to higher frequency, while *f*_*cl*_ keeps constant when *n* increases, as shown in Fig. 5(b).

The above results reveal that *f*_*cl*_ is determined by the total widths of the ERSCS, whereas *f*_*ch*_ is determined by *d* or PN which can also be written as PN = *c*_0_/2*dgf*, i.e., the resolution of the phase surface. Noting that for different *n*, *f*_*ch*_ is always smaller than the cutoff frequency of the waveguide *f*_c_ *=* *c*_0_/2*d*_0_ (e.g., for *n* *=* 18, *f*_*ch*_ *=* 20.1KHz and *f*_*c*_ *=* 22.9kHz). Hence, a sufficiently low discrete resolution will give rise to undesired reflected wave, which determines the value of *f*_*ch*_. In Figs. 5(a,b), upper 3 dB cutoff frequency is approximately PN *=* 2.41. For an extreme case of PN *=* 2, which means the phase oscillates between 0 to *π*, the phase changes are symmetric between *x* to −*x* and −*x* to *x*, leading to two symmetry reflected directions with corresponding angles of ±45°, as shown in Figs. 5(c, d). Thus, in Fig. 5(a), the value of 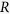 at PN *=* 2 is nearly half of the value of *R* at PN > 2.41 because only half of the scattered wave reflects to the desired region. Figures 5(e, f) show that a sidelobe appears at 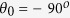 with PN *=* 2.41, which corresponds to 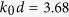, consistent with the result in Fig. 5(g). This spatial aliasing phenomenon and can be well explained by Eq. (9). 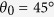 is one solution to the equation *D*(*θ*) *=* 1, corresponding to *k*_0_*d*(*sinθ*-2*g*)/2 = 0. Yet there is another solution, i.e., *k*_0_*d*(*sinθ*-2*g*)/2 *=* *mπ*(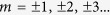). Generally such a condition is hard to satisfy since the discrete resolution is usually high and the value of *k*_0_*d*(*sinθ*-2*g*)/2 cannot reach ±*π*. When the frequency is high enough (viz., 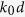 is sufficiently low), however, another solution corresponding to *k*_0_*d*(*sinθ*-2*g*)/2 =−*π* will appear. Figure 3(g) gives the relationship between the values of *K*_0_*d* and the extraordinary reflection angles when sidelobe appears at *θ*_0_ =−90°, which satisfies
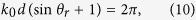
where *θ*_*r*_ is the extraordinary reflection angles. The numerical results agree well with the theoretical predictions, in which the value of *K*_0_*d* gives the upper cutoff frequency of extraordinary reflection. The above analyses suggest that for the purpose of mimicking the desired phase surface by using discrete units which necessarily requires a fine spatial resolution^20^, the simplified model developed here can be used to predict the upper cutoff frequency accurately. Thanks to the simple structure of the designed subwavelength corrugated surface, by just increasing *n* one can have extended bandwidth and finer resolution of the phase surface that provides a full freedom for wavefront control that will be applicable to any beam shaping.

### Realization of arbitrary dispersionless phase fronts

As we have discussed above, Eq. (3) implies that the general scheme proposed by us allows one to design arbitrary phase profile as a function of *x* while keeping it frequency-independent. For the particular implementation by the SCS made of subwavelength grooves, such potential could be realized by choosing the gradient of *h* as a more complicated function of *x*. Thereby any desired dispersionless phase front can be realized, such as planar focusing, and accelerating non-diffractive beam with arbitrary convex trajectory. In detail, if a spatial phase 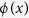 is needed, according to Eq. (4), the depth of groove should be chosen as
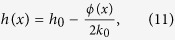
where 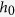 is a constant introduced for ensuring that *h*(*x*) is a positive value. Then the resulting phase front is dispersionless due to the fact that 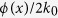 is independent of wavelength, and the resolution of phase profile can be controlled freely by adjusting the value of 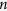.

### Planar focusing

In order to focus sound at an arbitrary position (*x*_0_, *y*_0_), a hyperboloidal phase profile is required
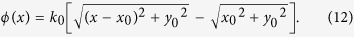


According to Eq. (12), the depths of grooves at different *x* locations are determined by
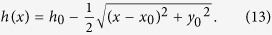


**Figure 6:**
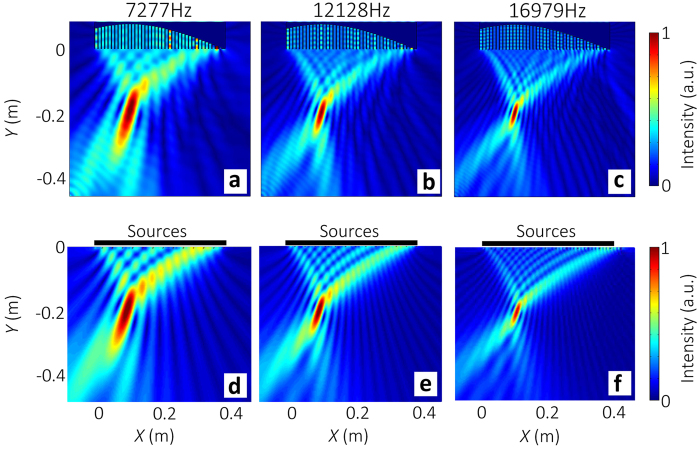
Illustration of sound focusing by SCS. The sample is made of 40 grooves with *d* = 1cm and *d*_0_ = 0.75cm. (a,b,c) Numerical results of the scattered acoustic intensity fields for normally-incident plane wave at frequencies of 7.277 kHz, 12.128 kHz, and 16.979 kHz, respectively. The focal location is (10 cm, −19 cm). (d, e, f) Acoustic intensity fields given by Eq. (6) at different frequencies.

Figures 6(a–c) show the scattered acoustic intensity fields of sound focusing model with the focal location (10 cm, −19 cm) at 7.277 kHz, 12.128 kHz and 16.979 kHz, respectively. The focusing phenomenon is obviously seen in ultra-broadband. The dispersionless focusing phenomenon can be also interpreted by phased array theory. Figures 6(d, e) display the acoustic intensity fields given by Eq. (6) based on the phased array theory, which agree with the numerical results. Here, *ϕ*_*j*_ in Eq. (6) is no longer linearly varying with *x*, but meets Eq. (12).

### Non-diffractive beam with arbitrary convex trajectory

We have also demonstrated SCS with generation of acoustic beam that propagates along arbitrary convex trajectory. On the basis of Caustic theory^21^,^22^, arbitrary convex accelerating beams can be obtained by directing an appropriate spatial phase profile. The relationship between the phase profile *ϕ*(*x*) and the reflected angle *θ* is 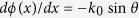. To realize an arbitrary trajectory 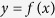, the phase profile can be expressed as^22^
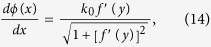
where 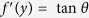 is the slope of the trajectory. As an example, a circular trajectory 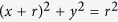 with 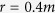 is designed. The slope of the curve at (*x*_0,_*y*_0_) is 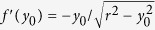. Owing to 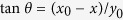 and 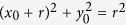, the slope of the trajectory can be expressed as
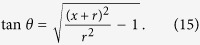


Combining Eq. (14), we obtain
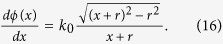


The spatial phase profile is
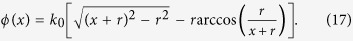


Thus, the depth *h*(*x*) is
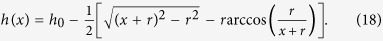


**Figure 7:**
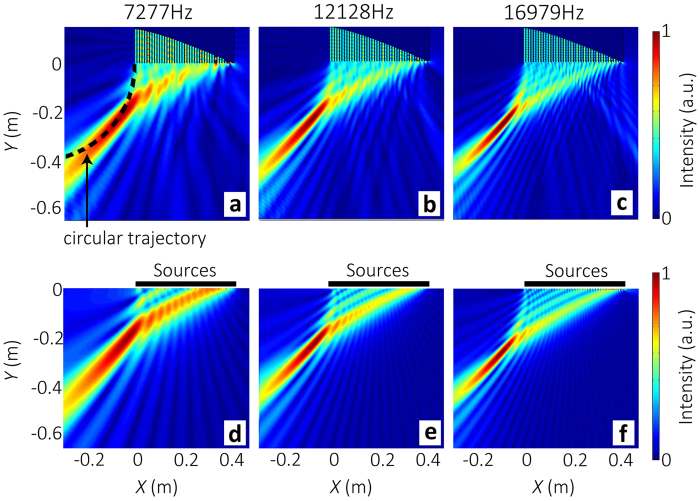
Illustration of non-diffractive beam with the circular trajectory by SCS. The sample is made of 40 grooves with *d* *=* 1cm and *d*_0_ *=* 0.75cm. (a, b, c) Numerical results of the scattered acoustic intensity fields for normally-incident plane wave at frequencies of 7.277 kHz, 12.128 kHz, and 16.979 kHz, respectively. The trajectory is (*x* + *r*)^2^ + *y*^2^ = *r*^2^ (dashed line). (d, e, f) Acoustic intensity fields given by Eq. (6) at different frequencies.

Figures 7(a–c) show the scattered acoustic intensity fields at 7.277 kHz, 12.128 kHz and 16.979 kHz, respectively. Figures 7(d, e) display the acoustic intensity fields given by Eq. (6), which is in good agreement with the numerical results.

## Discussion

Realization of dispersionless manipulation of wavefront is a challenge in optic/acoustic wave steering. We have proposed a general scheme for resolving this challenge and showed that a simple corrugated surface with subwavelength grooves can freely control the reflected waves to form the desired wave field without frequency limitation. An analytical model is developed to interpret this extraordinary phenomenon and can be used to predict the upper cutoff frequency when using a discrete phase array to mimick a continuous phase profile^2^. On the other hand, the proposed reflective structure has the potential to be combined with a waveguide structure for realizing unconventional wave-steering effect in waveguides^23^,^24^. Our finding may pave the way to design of ultra-broadband optical/acoustical wavefront-devices, and have potential applications in a variety of practical situations in need of special harness of acoustic wave such as medical ultrasound application or field caustics engineering. The simple configuration and distinct reflective characteristic of the proposed structure also makes it particularly inspiring in the fields of architectural or environmental acoustics generally associated with the control of reflection of broadband signals, and may result in conceptual devices such as flat walls capable of redirect and reshape sound at will.

## Methods

### Acoustic measurements

Our experiment was carried in the anechoic chamber in order to eliminate the influence of the reflected waves. An absorptive plate was used to separate the incident and reflected acoustic fields as shown in Fig. 2(c). To obtain an acoustic plane wave, a loudspeaker was located 3 m away from the sample (much larger than the width of the sample). The measuring area and the center of loudspeaker are located in the same *x-y* plane. Two 0.25-inch-diameter Bruel & Kjær type-4961 microphones are used to detect the acoustic pressure, one is used to scan the measuring region, and another is fixed near the loudspeaker to detect the phase information.

### Numerical simulations

Numerical simulations are conducted with COMSOL Multiphysics software. The simulated material for ABS has a density 1180 kg/m3 and sound speed 2700 m/s, which are the parameters of the 3D-printed materials in the experiments. The surrounding medium is air with its density 1.21 kg/m^3^ and sound speed 343 m/s. The viscous effect has been ignored in our simulations, corresponding to the experimental situation, where the thickness of viscous boundary layer, approximating to 0.3 mm for lower frequency limitation, is about 25 times smaller than the groove width.

## Additional Information

**How to cite this article**: Zhu, Y.-F. *et al.* Dispersionless Manipulation of Reflected Acoustic Wavefront by Subwavelength Corrugated Surface. *Sci. Rep.* **5**, 10966; doi: 10.1038/srep10966 (2015).

## Supplementary Material

Supplementary Information
