# Supplementary material for: Dispersionless Manipulation of Reflected Acoustic Wavefront by Subwavelength Corrugated Surface
Source: Sci Rep. 2015 Jun 16;5:10966. doi: 10.1038/srep10966 (PMC4468515; doi:10.1038/srep10966)
Supplement: Supplementary Information [file srep10966-s1.pdf]

Supplementary Materials of

"Dispersionless Manipulation of Reflected Acoustic Wavefront by Subwavelength Corrugated Surface"

Yi-Fan Zhu[1,2†], Xin-Ye Zou[1,2†], Rui-Qi Li[1], Xue Jiang[1], Juan Tu[1], Bin Liang[1,2*], and Jian-Chun Cheng[1,2,3*]

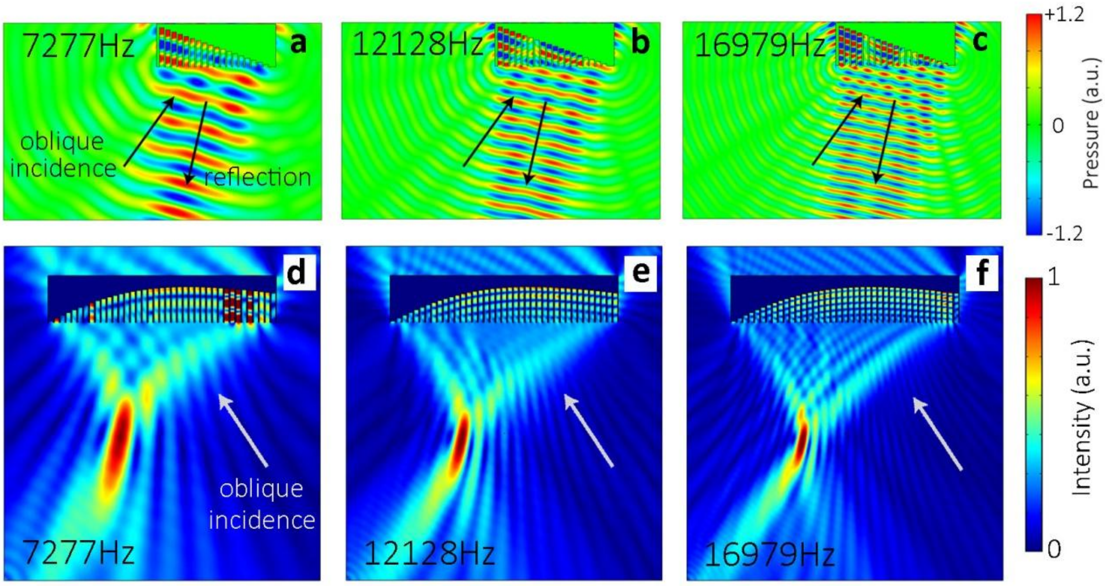

Figure S1 | Simulations for oblique incident cases. (a-c) Extraordinary reflection with $-30°$ incidence at 7.277kHz, 12.128kHz, and 16.979kHz, respectively. (d-f) planar focusing with $30°$ incidence at 7.277kHz, 12.128kHz, and 16.979kHz, respectively.

Due to the fact that width of groove (0.75cm), despite its subwavelength scale in terms of the working wavelength, is much larger than the thickness of the tube wall

(0.25cm), it is expectable that the extraordinary phenomena（extraordinary reflection, focusing and more complicated phase profiles）with high efficiency will also be observed for oblique incidence. Figures S1(a-c) show the extraordinary reflection with -30° incidence at 7.277kHz, 12.128kHz, and 16.979kHz, respectively. Figures S1(d-f) show the planar focusing with 30° incidence at 7.277kHz, 12.128kHz, and 16.979kHz, respectively. A phase compensation is added to realize planar focusing for the oblique incident cases.
